# Crude extract of *Polygonum cuspidatum* stimulates immune responses in normal mice by increasing the percentage of Mac-3-positive cells and enhancing macrophage phagocytic activity and natural killer cell cytotoxicity

**DOI:** 10.3892/mmr.2014.2739

**Published:** 2014-10-22

**Authors:** FU-SHIN CHUEH, JEN-JYH LIN, JU-HWA LIN, SHU-WEN WENG, YI-PING HUANG, JING-GUNG CHUNG

**Affiliations:** 1 Department of Health and Nutrition Biotechnology, Asia University, Taichung, Taiwan, R.O.C.; 2 School of Chinese Medicine, China Medical University, Taichung, Taiwan, R.O.C.; 3 Division of Cardiology, China Medical University Hospital, Taichung, Taiwan, R.O.C.; 4 Department of Biological Science and Technology, China Medical University, Taichung, Taiwan, R.O.C.; 5 Department of Chinese Medicine, Taichung Hospital, Department of Health, Executive Yuan, Taichung, Taiwan, R.O.C.; 6 Department of Physiology, China Medical University, Taichung, Taiwan, R.O.C.; 7 Department of Biotechnology, Asia University, Taichung, Taiwan, R.O.C.

**Keywords:** crude extract of *Polygonum cuspidatum*, BALB/c mice, phagocytosis, macrophage, natural killer cells

## Abstract

*Polygonum cuspidatum* is a natural plant that is used in traditional Chinese herbal medicine. The crude extract of *Polygonum cuspidatum* (CEPC) has numerous biological effects; however, there is a lack of studies on the effects of CEPC on immune responses in normal mice. The aim of the present study was to determine the *in vivo* effects of CEPC on immune responses in normal mice. CEPC (0, 50, 100, 150 and 200 mg/kg) was orally administered to BALB/c mice for three weeks, following which blood, liver, and spleen samples were collected. CEPC did not significantly affect the total body weight, or tissue weights of the liver or spleen, as compared with the control mice. CEPC increased the percentages of CD3 (T-cell marker), 11b (monocytes) and Mac-3 (macrophages) positive-cells, and reduced the percentage of CD19-positive cells (B-cell marker), as compared with the control mice. CEPC (100 mg/kg) stimulated macrophage phagocytosis of blood samples but did not affect macrophage phagocytosis in the peritoneum. Activity of the splenic natural killer cells was increased in response to CEPC (50 mg/kg) treatment. Furthermore, CEPC inhibited T- and B-cell proliferation when the cells were stimulated with concanavalin A and lipopolysaccharide, respectively.

## Introduction

Numerous studies have previously shown that fruit and vegetable consumption may reduce the risk of developing cancers of the oropharynx, oesophagus, lung, stomach and colorectum (1,2). Furthermore, it may also reduce the risk of oxidative stress and cell damage (3), cardiovascular diseases and atherosclerosis (4). Due to the safety, low toxicity, reduced side effects and general availability, phytochemicals and dietary compounds have been used for the treatment of human cancer (5). White blood cells interact with each other to produce an immune response against specific antigens (6). It has been well documented that increasing the immune response will improve the defense against various diseases, microbial infections and leukemia (7). Therefore, research has focused on the identification of novel compounds from plants, which may promote the immune response.

*Polygonum cuspidatum* is widely distributed in southern China and Japan. The root of *Polygonum cuspidatum* has previously been used to treat inflammation, infection and hyperlipidemia (8). Emodin is isolated from *Polygonum cuspidatum* and has numerous biological effects. Emodin has previously been shown to inhibit Coxsakievirus B4 *in vitro* and *in vivo* (9), and numerous studies have reported that emodin possesses an anticancer function (10–12). However, there is currently no available information on the effects of *Polygonum cuspidatum* on the immune responses of normal mice *in vivo*.

The present study aimed to investigate the effects of the crude extract of *Polygonum cuspidatum* (CEPC) on the immune responses of normal BALB/c mice *in vivo*.

## Materials and methods

### Materials and reagents

Dimethyl sulfoxide (DMSO) was obtained from Sigma-Aldrich (St. Louis, MO, USA). RPMI-1640 medium, fetal bovine serum, L-glutamine and penicillin-streptomycin were obtained from Gibco Life Technologies (Carlsbad, CA, USA). CEPC, provided by Dr Fu-Shin Chueh (Department of Health and Nutrition Biotechnology, Asia University, Taichung, Taiwan), was dissolved in DMSO at 1% and stored at −20°C, in a 50 ml tube covered with aluminum, until further use.

### Male BALB/c mice

A total of 50 male BALB/c mice, 8 weeks old and weighing 22–25 g, were obtained from the National Laboratory Animal Center (Taipei, Taiwan). The mice were maintained in specified pathogen-free conditions in the animal center of the China Medical University (Taichung, Taiwan). The mice were monitored and received a normal diet. The use of mice in the present study was approved by the Institutional Animal Care and Use Committee of the China Medical University (Taichung, Taiwan), as previously described (13).

### In vivo treatment of animals with CEPC

A total of 50 male BALB/c mice were randomly divided into five groups (10 mice/group): Group I mice were treated with a normal diet and served as a control group; group II mice were treated with 25 mg/kg CEPC; group III mice were treated with 50 mg/kg CEPC; group IV mice were treated with 100 mg/kg CEPC; and group V mice were treated with 200 mg/kg CEPC. The CEPC was mixed with olive oil and was administered daily by oral gavage, at the indicated doses, for 27 days. At the end of the treatment, all of the mice were weighed and sacrificed by euthanasia, performed by delivering increasing concentrations of CO_2_, as previously described (14).

### Immunofluorescence staining of the surface markers of immune cells from each mouse

The mice were weighed following 27 days of CEPC treatment. Blood samples were then collected by cardiac puncture, and the spleens were harvested. The splenocytes were isolated to measure natural killer (NK) cell activity. To determine the number of leukocyte cells, 1 ml blood was collected from the mice and lysed using 1× Pharm Lyse™ lysing buffer (BD Biosciences, Franklin Lakes, NJ, USA). The samples were centrifuged at 1,500 × g, for 15 min at 4°C, in order to collect the white blood cells. The leukocytes were stained with phycoerythrin (PE)-labeled anti-mouse CD3 (1:100; catalog number, 553062; clone, 145-2C11), PE-labeled anti-mouse CD19 (1:100; catalog number, 561740; clone, 1D3), fluorescein isothiocyanate (FITC)-labeled anti-mouse CD11b (1:100; catalog number, 57396; clone, M1/70) and Mac-3 (1:100; catalog number, 553324; clone, M3/84) antibodies (BD Pharmingen, San Diego, CA, USA), for 30 min at 4°C and were then washed with phosphate-buffered saline (PBS). The cells were then stained with a secondary antibody and analyzed by flow cytometry to determine the percentage of cell markers, as described by previous methods (14).

### Quantification of macrophage phagocytic activity

Macrophages were isolated from the peripheral blood mononuclear cells (PBMC) and the peritoneum of the mice. The isolated macrophages were placed in a fluorescence-activated cell sorting tube and 50 μl *E. coli*-FITC was added, according to the manufacturer’s instructions of the PHAGOTEST^®^ kit (ORPEGEN Peptide Chemicals GmbH, Heidelberg, Germany), and as previously described (14) The samples were analyzed using flow cytometery and quantified using CellQuest software (BD Biosciences), as previously described (14,15).

### Quantification of NK cell cytotoxic activity

The isolated splenocytes (1×10^5^ cells) were placed in each well of a 96-well plate in 50 μl RPMI-1640 medium. YAC-1 mouse lymphoma cells (2.5×10^7^ cells; Bioresource Collection and Research Center, Hsinchu, Taiwan) in serum-free RPMI 1640 medium and the PKH-67/Dil.C buffer (Sigma-Aldrich) were then added to the cells and mixed thoroughly, for 2 min at 25°C. A total of 50 μl PBS was added to each well for 1 min, followed by 100 μl medium and incubated for 10 min. The cells were then centrifuged at 1,200 × g, for 2 min at 25°C. NK cell cytotoxic activity was determined by flow cytometry, as previously described (14,15).

### Determination of T- and B-cell proliferation

The isolated splenocytes (1×10^5^ cells/well) were placed in a 96-well plate. A total of 100 μl RPMI-1640 medium was added to each well, and the cells were stimulated with concanavalin A (Con A, 5 μg/ml) for three days to initiate T-cell proliferation, and with lipopolysaccharide (LPS, 5 μg/ml), for 5 days to initiate B-cell proliferation. All of the samples were measured using the CellTiter 96 AQueous One Solution Cell Proliferation Assay kit (Promega Corporation, Madison, WI, USA), as previously described (13,15).

### Statistical analysis

All of the experiments in the present study were repeated at least three times. The data were expressed as the means ± standard deviation. Comparisons between the control and CEPC-treated groups were analyzed by student’s t-test. A P<0.05 was considered to indicate a statistically significant difference.

## Results

### Effects of CEPC on the body and organ weights of BALB/c mice

**Figure 1:**
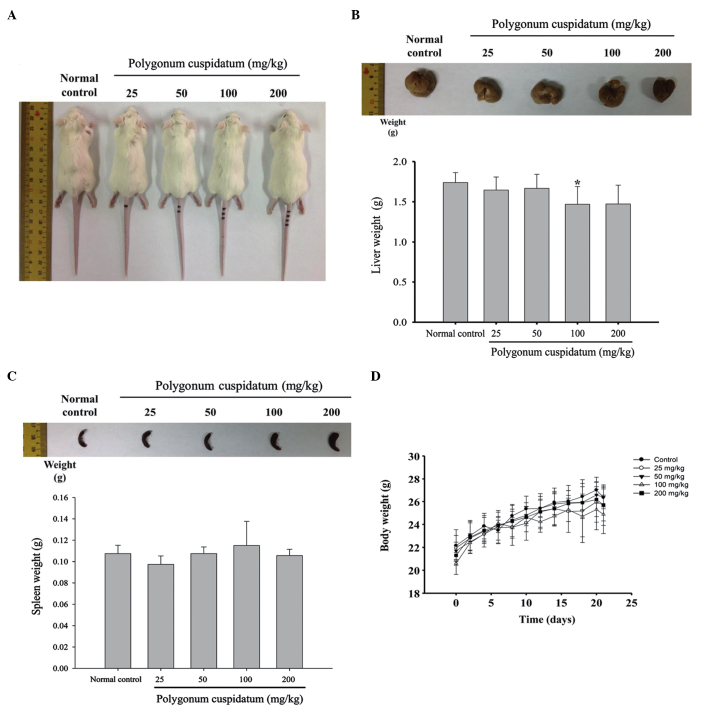
Effects of crude extract of *Polygonum cuspidatum* (CEPC) on the body, liver and spleen weights of normal BALB/c mice. The mice were divided into five groups, which were treated with different concentrations of CEPC: Group I, 0 mg/kg CEPC (control); group II, 25 mg/kg CEPC; group III, 50 mg/kg CEPC; group IV 100 mg/kg CEPC; group V 200 mg/kg CEPC. All of the mice were treated with CEPC for 27 days. (A) Representative images of the mouse body. The weights of the (B) liver, (C) spleen and (D) total body, which was weighed every three days. The data are expressed as the means ± standard deviation, of three experiments.

The mice were administered CEPC (25, 50, 100, 200 mg/kg), or normal control, for 27 days. Every three days the mice were weighed, and the murine tissues were weighed at the end of the CEPC treatment (Fig. 1A–D). CEPC administration, at any of the four doses, did not significantly alter body, liver or spleen weight, as compared with the control mice.

### Effects of CEPC on leukocyte cell markers in BALB/c mice

**Figure 2:**
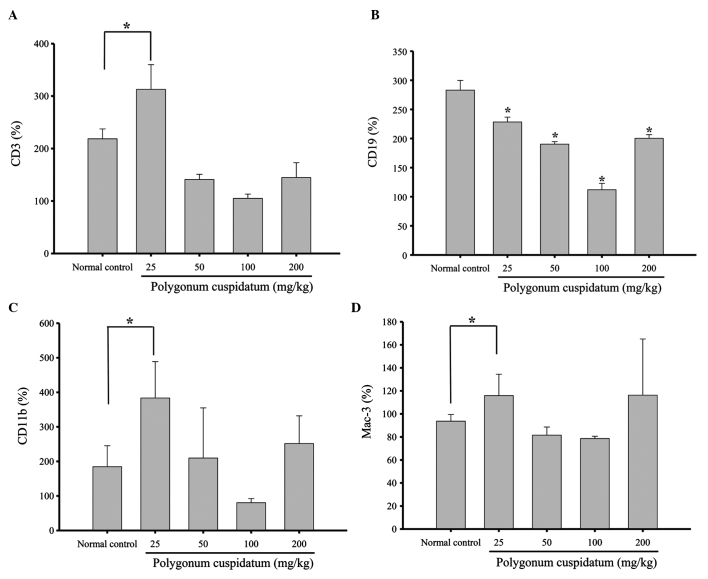
Effects of crude extract of *Polygonum cuspidatum* (CEPC) on the levels of white blood cell markers from normal BALB/c mice. The blood was collected from CEPC-treated and control mice and analyzed for cell markers: (A) CD3; (B) CD19; (C) CD11b and (D) Mac-3, by flow cytometry. The data are expressed as the means ± standard deviation, of three experiments (n=10). ^*^P<0.05, the difference between control and CEPC-treated groups.

Flow cytometry was performed to measure the levels of cell markers CD3, CD19, CD11b and Mac-3, in the CEPC-treated and control mice. CEPC treatment (25 mg/kg) increased the levels of CD3 (Fig. 2A), CD11 (Fig. 2C) and Mac-3 (Fig. 2D); however, the levels of CD19 were decreased (Fig. 2C) in response to 25, 50, 100 and 200 mg/kg CEPC treatment, as compared with the control group. These results demonstrate that CEPC significantly affects the white blood cell proliferation of normal mice *in vivo*.

### Effects of CEPC on macrophage phagocytic activity from the PBMC and peritoneal cavity of BALB/c mice

**Figure 3:**
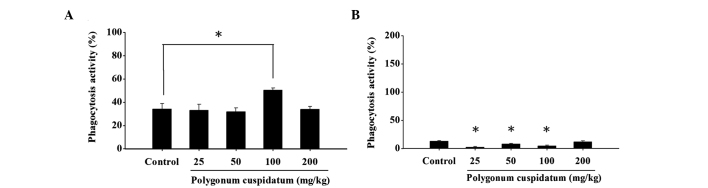
Effects of crude extract of *Polygonum cuspidatum* (CEPC) on the phagocytic activity of macrophages isolated from (A) peripheral blood mononuclear cells and (B) peritoneal cavity of normal BALB/c mice, as measured by flow cytometery and quantified by CellQuest. The data are expressed as the means ± standard deviation. ^*^P<0.05, the difference between control and CEPC-treated groups.

The macrophages were isolated from the PBMC and peritoneal cavity, and the levels of phagocytosis were analyzed by flow cytometry. Treatment with CEPC, at all four doses, significantly reduced macrophage phagocytosis from the PBMC (Fig. 3A). Conversely, the macrophage phagocytotic activity was not significantly stimulated in the cells from the peritoneal cavity at a CEPC dose of 25, 50 or 100 mg/kg, as compared with the control mice (Fig. 3B).

### Effects of CEPC on the cytotoxic activity of NK cells and B- and T-cell proliferation in BALB/c mice

**Figure 4:**
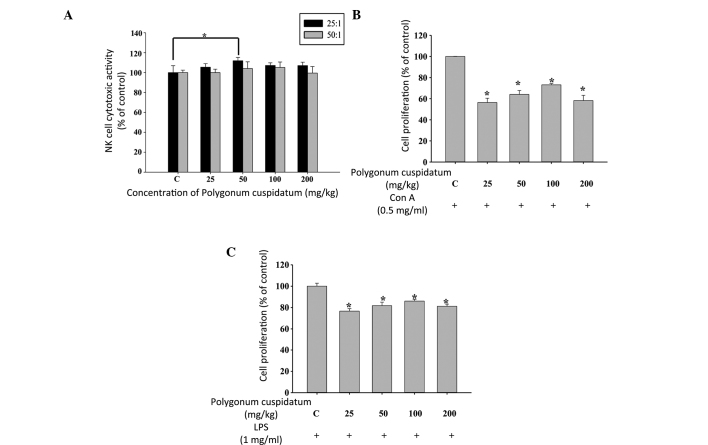
Effects of crude extract of *Polygonum cuspidatum* (CEPC) on the cytotoxic activity of natural killer (NK) cells and T- and B-cell proliferation from normal BALB/c mice. (A) Isolated splenocytes (1×10^5^ cells/well) were placed in 1 ml of RPMI-1640 medium in 96-well plates. Target YAC-1 mouse lymphoma cells (2.5×10^7^) in serum-free RPMI-1640 medium and the PKH-67/Dil.C buffer was added to the cells for the determination of the NK cell cytotoxic activity by flow cytometry. (B) B-cells were pretreated with lipopolysaccharide (LPS) and proliferation was analyzed by flow cytometry. (C) T-cells were pretreated with concanavalin A (Con A) and cell proliferation was analyzed by flow cytometry. The data are expressed as the means ± standard deviation. ^*^P<0.05, a difference between the control and CEPC-treated groups.

The YAC-1 target cells were destroyed by the NK cells, which were isolated from the splenocytes of mice treated with 50 mg/kg CEPC (Fig. 4A). However, the other CEPC doses did not alter the NK activity. Treatment with 25 mg/kg CEPC increased both B- (Fig. 4B) and T-cell (Fig. 4C) proliferation. However, CEPC doses of 100 and 150 mg/kg did not significantly alter the proliferation of B- and T-cells.

## Discussion

There are currently few reports on the biological effects of CEPC, including its antiviral, antimicrobial, and cardioprotective activities (16), and no studies have examined the effects of CEPC on immune responses *in vivo*. The present study examined the effects of CEPC on immune responses in BALB/c mice *in vivo*. The mice were treated with or without CEPC at various doses (50, 100, 150 and 200 mg/kg). CEPC treatment did not alter the body weight of the mice, as compared with the control mice, and liver and spleen weights were not altered by CEPC treatment. CEPC, at the cellular level, altered immune responses, including increased proliferation of T- and B-cells, and increased the levels of monocyte and macrophage markers. CEPC was also shown to promote the phagocytic activities of macrophages, and enhance the cytotoxic effects of NK cells. Furthermore, 25 mg/kg CEPC treatment promoted and enhanced the populations of CD3, CD11b and Mac-3-positive cells; however, no significant effects were observed in response to the higher doses of CEPC (50, 100 and 200 mg/kg). Conversely, all of the doses of CEPC (25, 50, 100 and 200 mg/kg) significantly decreased the population of CD19-positive cells, CD19 is an activated B-cell surface marker (17,18).

Treatment with CEPC increased the number of cells positive for the T-cell marker CD3. T-cells are involved in cell-mediated immune responses (14,19). Deletion of T-cells in animals has been shown to result in the loss of cellular and humoral immune responses (20). This is the case in patients with acquired immunodeficiency syndrome, where the loss of T-cells is due to the destruction of T helper cells by the human immunodeficiency virus (19). CD11b and Mac-3 are markers of monocytes and macrophages, respectively. Both of these cell markers were increased in response to CEPC (50 mg/kg), which may be indicative of increased phagocytic activity of the macrophages.

Previous studies have demonstrated that antigens induce macrophage activity, including phagocytosis and stimulation of T-cell functions, including cytotoxic and helper T-cells. Activated T-cells release cytokines which also promote macrophage function (21,22). Macrophages can suppress intracellular bacterial growth and lead to a reduction in infection (23).

Treatment with 100 mg/kg CEPC promoted the phagocytic activity of macrophages isolated from the PBMC; however, the other doses of treatment did not result in any significantly promoted activities. A treatment with 50 mg/kg CEPC promoted NK cell activities from the spleen samples, but other doses of CEPC treatment did not show any significant promoted activities of the NK cells.

Furthermore, CEPC treatment at all of the indicated doses resulted in a decrease in T- and B-cell proliferation, following Con A and LPS stimulation, respectively. However, further investigations are required. The results of the present study demonstrated that CEPC may enhance the population of Mac-3-positive cells and promote the phagocytic activity of macrophages.

In conclusion, it may be suggested that CEPC may stimulate proliferation of monocytes (CD11b) and enhance macrophage (Mac-3) function, including phagocytosis, *in vivo*. Furthermore, CEPC promoted NK cell activities, which may be associated with the increased levels of T-, monocyte and macrophage cell surface markers in normal BALB/c mice *in vivo*.

## References

[1] Soerjomataram I, Oomen D, Lemmens V (2010). Increased consumption of fruit and vegetables and future cancer incidence in selected European countries. Eur J Cancer.

[2] Tsai CW, Chen HW, Sheen LY, Lii CK (2012). Garlic: Health benefits and actions. BioMedicine.

[3] Guizani N, Waly MI, Singh V, Rahman MS (2013). Nabag (*Zizyphus spina-christi*) extract prevents aberrant crypt foci development in colons of azoxymethane-treated rats by abrogating oxidative stress and inducing apoptosis. Asian Pac J Cancer Prev.

[4] Toh JY, Tan VM, Lim PC, Lim ST, Chong MF (2013). Flavonoids from fruit and vegetables: a focus on cardiovascular risk factors. Curr Atheroscler Rep.

[5] Pratheeshkumar P, Sreekala C, Zhang Z (2012). Cancer prevention with promising natural products: mechanisms of action and molecular targets. Anticancer Agents Med Chem.

[6] Yu FS, Yang JS, Yu CS (2013). Safrole suppresses murine myelomonocytic leukemia WEHI-3 cells in vivo, and stimulates macrophage phagocytosis and natural killer cell cytotoxicity in leukemic mice. Environ Toxicol.

[7] Paul DJ, Laure NB, Guru SK (2014). Antiproliferative and antimicrobial activities of alkylbenzoquinone derivatives from *Ardisia kivuensis*. Pharm Biol.

[8] Peng W, Qin R, Li X, Zhou H (2013). Botany, phytochemistry, pharmacology, and potential application of *Polygonum cuspidatum* Sieb.et Zucc.: a review. J Ethnopharmacol.

[9] Liu Z, Wei F, Chen LJ (2013). In vitro and in vivo studies of the inhibitory effects of emodin isolated from *Polygonum cuspidatum* on Coxsakievirus B_4_. Molecules.

[10] Wei WT, Lin SZ, Liu DL, Wang ZH (2013). The distinct mechanisms of the antitumor activity of emodin in different types of cancer (Review). Oncol Rep.

[11] Ma YS, Weng SW, Lin MW (2012). Antitumor effects of emodin on LS1034 human colon cancer cells in vitro and in vivo: roles of apoptotic cell death and LS1034 tumor xenografts model. Food Chem Toxicol.

[12] Chang YC, Lai TY, Yu CS (2011). Emodin induces spoptotic death in murine myelomonocytic leukemia WEHI-3 cells in vitro and enhances phagocytosis in leukemia mice in vivo. Evid Based Complement Alternat Med.

[13] Tan TW, Lin YT, Yang JS (2009). *A. cantoniensis* inhibits the proliferation of murine leukemia WEHI-3 cells in vivo and promotes immunoresponses in vivo. In Vivo.

[14] Lin CC, Yu CS, Yang JS (2012). Chrysin, a natural and biologically active flavonoid, influences a murine leukemia model in vivo through enhancing populations of T- and B-cells, and promoting macrophage phagocytosis and NK cell cytotoxicity. In Vivo.

[15] Tsou MF, Tien N, Lu CC (2013). Phenethyl isothiocyanate promotes immune responses in normal BALB/c mice, inhibits murine leukemia WEHI-3 cells, and stimulates immunomodulations in vivo. Environ Toxicol.

[16] Zhang H, Li C, Kwok ST, Zhang QW, Chan SW (2013). A review of the pharmacological effects of the dried root of *Polygonum cuspidatum* (Hu Zhang) and its constituents. Evid Based Complement Alternat Med.

[17] Asano N, Fujimoto M, Yazawa N (2004). B Lymphocyte signaling established by the CD19/CD22 loop regulates autoimmunity in the tight-skin mouse. Am J Pathol.

[18] Jarasch-Althof N, Wiesener N, Schmidtke M, Wutzler P, Henke A (2010). Antibody-dependent enhancement of coxsackievirus B3 infection of primary CD19+ B lymphocytes. Viral Immunol.

[19] Bixler SL, Mattapallil JJ (2013). Loss and dysregulation of Th17 cells during HIV infection. Clin Dev Immunol.

[20] Porichis F, Hart MG, Zupkosky J, Barblu L, Kaufmann DE (2013). In vitro assay to evaluate the impact of immunoregulatory pathways on HIV-specific CD4 T cell effector function. J Vis Exp.

[21] Bhardwaj N, Nash TW, Horwitz MA (1986). Interferon gamma-activated human monocytes inhibit the intracellular multiplication of *Legionella pneumophila*. J Immunol.

[22] Nash TW, Libby DM, Horwitz MA (1988). IFN-gamma-activated human alveolar macrophages inhibit the intracellular multiplication of *Legionella pneumophila*. J Immunol.

[23] Horwitz MA (1983). Cell-mediated immunity in Legionnaires’ disease. J Clin Invest.

